# CpG ODN and ISCOMATRIX Adjuvant: A Synergistic Adjuvant Combination Inducing Strong T-Cell IFN-*γ* Responses

**DOI:** 10.1155/2013/636847

**Published:** 2013-03-23

**Authors:** Michael J. McCluskie, Risini D. Weeratna, Dana M. Evans, Shawn Makinen, Debbie Drane, Heather L. Davis

**Affiliations:** ^1^Pfizer Vaccine Research, Ottawa Laboratories, 340 Terry Fox Drive, Suite 200, Ottawa, ON, Canada K2K 3A2; ^2^CSL Limited, 45 Poplar Road, Parkville, VIC 3052, Australia

## Abstract

For the induction of robust humoral and cellular immune responses, a strong rationale exists to use vaccine-adjuvant combinations possessing both immune modulatory and enhanced delivery capabilities. Herein, we evaluated the combination of 2 different adjuvants, a TLR9 agonist, composed of synthetic oligodeoxynucleotides (ODN) containing immunostimulatory CpG motifs (CpG), and ISCOMATRIX adjuvant (ISCOMATRIX), composed of saponin, phospholipid, and cholesterol, which possesses both immunostimulatory and delivery properties. While both individual adjuvants have been shown effective in numerous preclinical and clinical studies, it is likely that for optimal adjuvant activity a combined adjuvant approach will be necessary. Herein, using three different antigens, namely, hepatitis B surface antigen (HBsAg), ovalbumin (OVA), and influenza A haemagglutinin antigen (HA), we show in mice that some adjuvant effects of CpG and ISCOMATRIX are further enhanced if they are used in combination. In particular, with all three antigens, IFN-*γ* levels were greatly increased with the CpG/ISCOMATRIX combination. The ability of the CpG/ISCOMATRIX combination to induce antitumor responses when administered with OVA following administration to mice of a highly metastatic OVA-secreting tumor cell line (B16-OVA melanoma) was also demonstrated. Thus the CpG/ISCOMATRIX combination may prove to be a valuable tool in the development of novel or improved vaccines.

## 1. Introduction

A major impediment in the development of novel vaccines has been the lack of safe yet effective vaccine adjuvants. In recent years, synthetic oligodeoxynucleotides containing CpG motifs (CpG) have gained considerable interest as vaccine adjuvants owing to their inherent ability to induce and enhance Th1-type immunity. Through their direct interaction with Toll-like receptor 9 (TLR9) on human B cells and dendritic cells (DC), as well as indirect effects on other immune cells such as monocytes, macrophages, and T cells, CpG enhance antigen presentation and induce the production of high levels of Th1 cytokines, resulting in the production of potent antigen-specific Th1-type immune responses [1]. CpG have been shown to enhance both humoral and cellular immunity are multiple species including humans [2–11]. These effects can be further enhanced if CpG are used in combination with other adjuvants possessing either immunomodulatory activity (e.g., QS21) [12, 13] or antigen delivery capability (e.g., liposomes and emulsions) [12, 14, 15]. Therefore, a combinatorial approach may be required to stimulate even stronger immune responses (in particular Th1-biased immunity) and, in the case of CpG as adjuvant, optimal adjuvanticity may be obtained if CpG is combined with another adjuvant possessing both immunomodulatory and enhanced delivery capabilities. This may be particularly true for effective control of cancer and chronic viral infection, where strong, broad, and multispecific Th1-type responses are required involving both CD4^+^ and CD8^+^T cell responses that depend on the induction of IFN-*γ*. 

Herein, we evaluated the combination of CpG with ISCOMATRIX adjuvant (ISCOMATRIX). ISCOMATRIX is composed of ISCOPREP saponin (purified fraction of *Quillaja * saponin), cholesterol, and phospholipid and possesses both immunomodulatory and antigen delivery capabilities. ISCOMATRIX can induce strong humoral and cellular immune responses in both cancer and chronic infectious disease vaccines in multiple species including humans [16–20]. ISCOMATRIX has a broad range of effects including enhancement of antigen delivery and facilitation of antigen presentation to antigen-presenting cells such as DCs, induction of DC maturation, recruitment of immune cells to the draining lymph nodes via cytokine and chemokine induction, and activation of both innate and adaptive immune systems [16, 21, 22].

The CpG/ISCOMATRIX combination was evaluated using 3 different antigens: hepatitis B surface antigen (HBsAg), chicken egg ovalbumin (OVA), and influenza haemagglutinin antigen (HA) and compared to either adjuvant alone. We also tested whether the CpG/ISCOMATRIX combination could induce antitumor responses when administered with OVA following administration to mice of a highly metastatic OVA-secreting tumor cell line (B16-OVA melanoma).

## 2. Materials and Methods

### 2.1. Antigens

HBsAg (recombinant protein from *Saccharomyces cerevisiae*, Seradyne, Indianapolis, IN), OVA (grade VII, Sigma-Aldrich, Saint Louis, MO), and HA (Texas 1/77 H3N2, from embryonated chicken eggs, Microbix Biosystems, Toronto, ON, Canada) were used.

### 2.2. Adjuvants

B Class CpG ODN with nuclease-resistant phosphorothioate backbone (Pfizer, Langenfeld, Germany) was used. ISCOMATRIX adjuvant (ISCOMATRIX) composed of ISCOPREP saponin (purified fraction from bark extract of the *Quillaja saponaria* tree), dipalmitoyl phosphatidylcholine, and cholesterol (CSL Limited, Victoria, Australia) was used. ISCOMATRIX and ISCOPREP are registered trademarks of ISCOTEC AB, a CSL company. Aluminum hydroxide (alum) was used in the form of Alhydrogel “85,” which was obtained from Brenntag Biosector (Denmark).

### 2.3. Immunizations

Female BALB/c mice (6–8 wk; *n* = 10/group) were used with HBsAg and HA, whereas female C57Bl/6 mice (6–8 wk; *n* = 10/group) were used with OVA. All vaccine formulations were made up to a total volume of 50 *μ*L with phosphate-buffered saline (PBS; Sigma Chemical Co., Saint Louis, MO) and administered by intramuscular (IM) injection in the left tibialis anterior (TA) muscle of mice lightly anaesthetized with Isoflurane (CDMV, Saint Hyacinthe, QC). Mice were immunized on days 0 and 28 with 1 *μ*g HBsAg, on days 0, 14, and 21 with 10 *μ*g OVA, or by a single immunization of 1 *μ*g HA. Antigens were administered alone or in combination with adjuvants. CpG, ISCOMATRIX, and alum were used at doses of 10, 1, and 25 *μ*g, respectively, whether used alone or in combination. Mouse strains, antigen, and adjuvant doses for each antigen were determined to be optimal based on previous studies conducted in our laboratories. All animal experiments were repeated on at least one independent occasion to ensure reproducibility of results.

### 2.4. Immune Assays

Animals were bled at various timepoints after immunization and antigen-specific total IgG, IgG1, and IgG2a (HBsAg and HA groups) or IgG2c (OVA groups) were measured in plasma by endpoint ELISA (in triplicate) for individual animals based on methods previously described [23], using 96-well plates coated with HBsAg (0.1 *μ*g/well), OVA (1 *μ*g/well), or HA (0.5 *μ*g/well). Spleens were removed aseptically and antigen-specific cytotoxic T lymphocyte (CTL) activity was measured (HBsAg and OVA groups) as previously described [24]. For this assay, lymphocytes were restimulated with irradiated murine cell line expressing HBsAg (P815/S) and OVA (EG7) for HBsAg and OVA immunized mice, respectively, or with nonantigen-expressing cells as controls.

IFN-*γ* secretion was measured in culture supernatants from antigen-restimulated splenocytes, obtained using HBsAg (5.0 *μ*g/mL), OVA (0.5 mg/mL), or HA (5.0 *μ*g/mL) as antigen-specific stimulants, either by ELISA (for HBsAg, OVA, and HA) as previously described [24] or by ELISPOT assay (for OVA). ELISPOT assay for IFN-*γ* used biotinylated antibodies, BD ELISPOT, as described by the manufacturer. IL-4 secretion was also measured by ELISA for HBsAg and HA antigens in culture supernatants taken at 24 hr using a commercially available kit (mouse IL-4 OpEIA; PharMingen, Mississauga, ON).

### 2.5. Murine Tumor Challenge Studies

To establish tumors, C57Bl/6 mice (*n* = 10/group) received 5 × 10^5^ live B16 melanoma cells expressing OVA (Dr. John Frelinger and Dr. Edith Lord; University of Rochester, NY) by IV injection. In this model B16-OVA cells metastasize rapidly to the lungs and then to other major organs typically resulting in death of the untreated animal within 30 days after tumor induction. Animals injected with PBS (unimmunized) were used as placebo controls. On days 7 and 14 after tumor challenge, mice were immunized with 50 *μ*g OVA alone or in combination with CpG, ISCOMATRIX, or the CpG/ISCOMATRIX combination. Five animals per group were euthanized 21 days after tumor induction to assess tumor burden and immunological readouts. Lungs were removed and metastases were counted manually using a dissecting microscope. The remaining animals were monitored for long-term survival.

All animal studies were conducted under approval of local Institutional Animal Care and Use Committees and in accordance with the guidelines of the Canadian Council on Animal Care (CCAC).

### 2.6. Statistical Analysis

Data were analyzed using GraphPad Prism (GraphPad Software, San Diego, CA). Statistical significance of the difference between the two groups was calculated by Student's 2-tailed *t*-test and between three or more groups by 1-factor analysis of variance (ANOVA) followed by post hoc analysis. Survival times following tumor challenges were compared using Kaplan-Meier curves and the log-rank (Mandel-Cox) test. Differences were considered to be not significant with *P* > 0.05.

## 3. Results

### 3.1. Effect of Different Adjuvants on Antigen-Specific Plasma IgG

**Figure 1:**
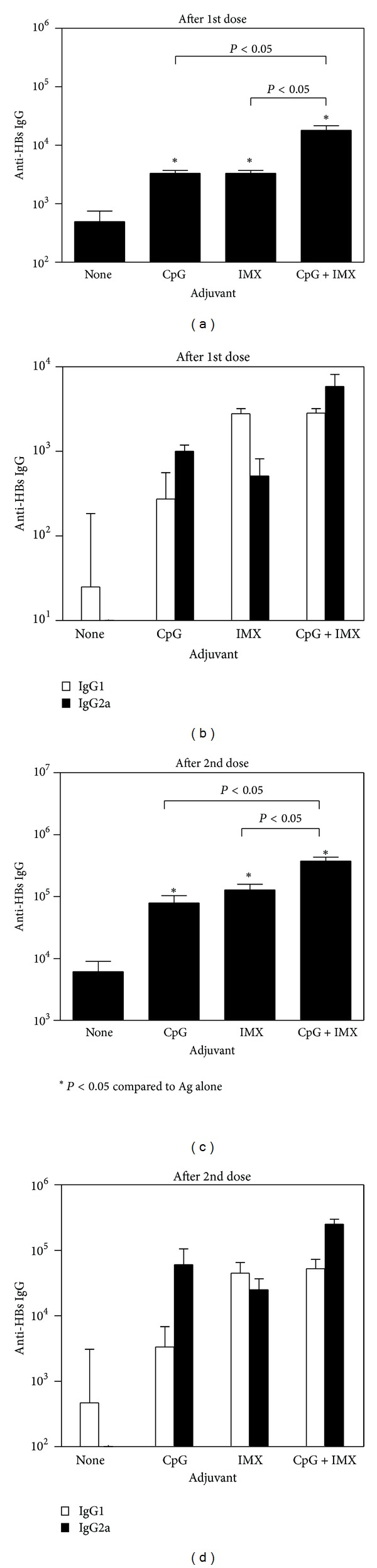
BALB/c mice (*n* = 10/gp) were immunized on days 0 and 28 by IM injection of 1 *μ*g HBsAg either alone (none) or combined with CpG (10 *μ*g), ISCOMATRIX (IMX) (1 *μ*g), or CpG (10 *μ*g) + ISCOMATRIX (1 *μ*g). (a) and (c) each bar represents the group geometric mean (±SEM) of the titer for HBsAg-specific antibodies (anti-HBs IgG) in plasma taken 4 wks after first (a) or second (c) immunization. (b) and (d) each bar represents the group geometric mean (±SEM) of the titer for HBsAg-specific antibodies (anti-HBs IgG) of IgG1 (open bars) or IgG2a (closed bars) in plasma taken 4 weeks after first (b) or second (d) immunization. Titers were defined as the highest plasma dilution resulting in an absorbance value two times that of nonimmune plasma with a cutoff value of 0.05. Representative data from one of five independent experiments is shown in each graph.

**Figure 2:**
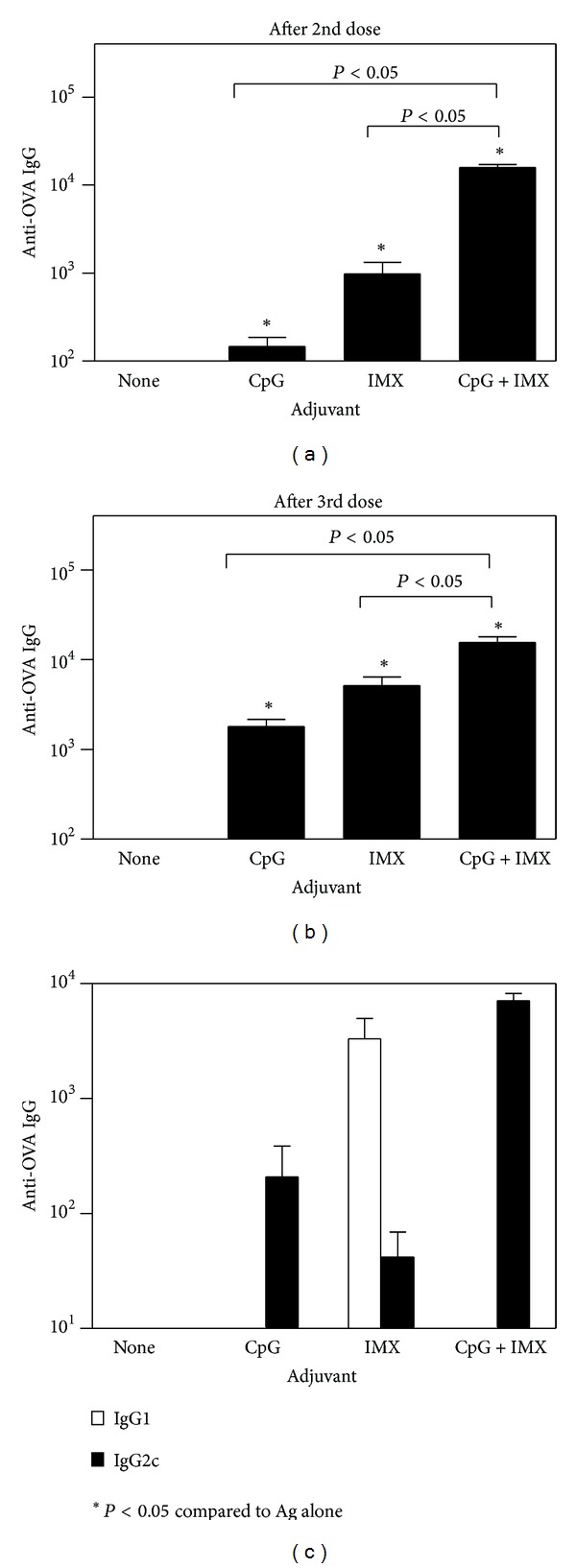
C57Bl/6 mice (*n* = 10/group) were immunized on days 0, 14, and 21 by IM injection of 10 *μ*g OVA either alone (none) or combined with CpG (10 *μ*g), ISCOMATRIX (IMX) (1 *μ*g), or CpG (10 *μ*g) + ISCOMATRIX (1 *μ*g). (a) and (b) each bar represents the group geometric mean (±SEM) of the titer for OVA-specific antibodies (anti-OVA IgG) in plasma taken 1 week after 2nd (a) or 3rd (b) immunization. (c) each bar represents the group geometric mean (±SEM) of the titer for OVA-specific antibodies (anti-OVA IgG) of IgG1 (open bars) or IgG2c (closed bars) in plasma taken 1 week after final immunization. Titers were defined as the highest plasma dilution resulting in an absorbance value two times that of nonimmune plasma with a cutoff value of 0.05. Representative data from one of five independent experiments is shown in each graph.

**Figure 3:**
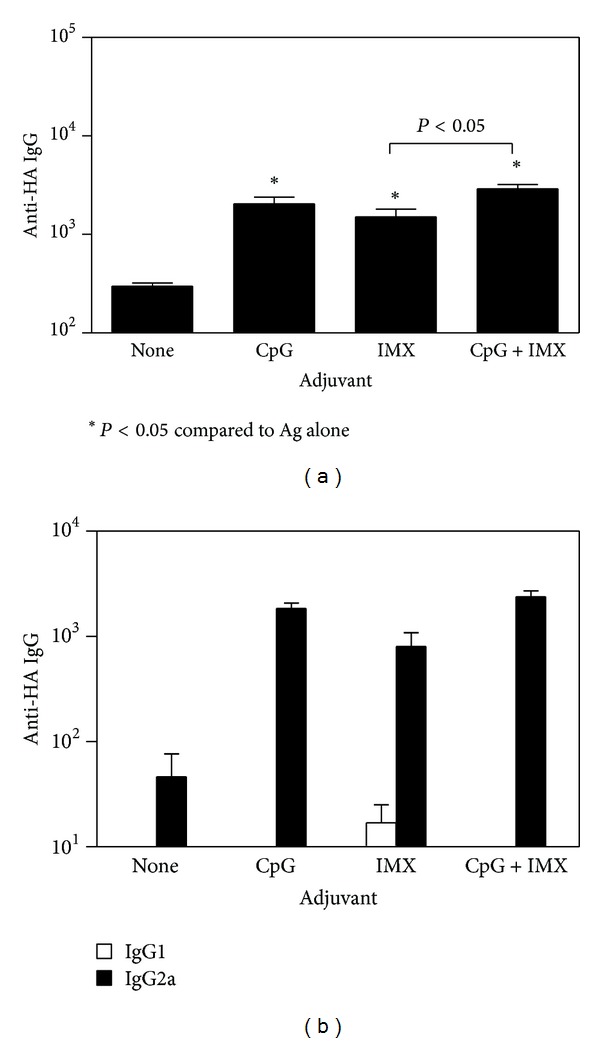
BALB/c mice (*n* = 10/group) received a single IM immunization of 1 *μ*g influenza. A haemagglutinin antigen (HA) either alone (none) or combined with CpG (10 *μ*g), ISCOMATRIX (IMX) (1 *μ*g), or CpG (10 *μ*g) + ISCOMATRIX (1 *μ*g). (a) each bar represents the group geometric mean (±SEM) of the titer for HA-specific antibodies (anti-HA IgG) in plasma taken 4 wks after immunization. (b) each bar represents the group geometric mean (±SEM) of the titer for HA-specific antibodies (anti-HA IgG) of IgG1 (open bars) or IgG2a (closed bars) in plasma taken 4 wks after immunization. Titers were defined as the highest plasma dilution resulting in an absorbance value two times that of nonimmune plasma with a cutoff value of 0.05. Representative data from one of two independent experiments is shown in each graph.

With all 3 antigens tested, antigen alone induced only low levels of Ag-specific IgG, even after boosting (Figures 1, 2, and 3). In contrast, the addition of either CpG or ISCOMATRIX significantly increased Ag-specific IgG levels (*P* < 0.05). With HBsAg (Figure 1) or OVA (Figure 2), levels of Ag-specific IgG were significantly higher with the CpG/ISCOMATRIX combination than with either adjuvant alone (*P* < 0.05). With HA (Figure 3), antibody levels with the CpG/ISCOMATRIX combination were higher than with ISCOMATRIX alone (*P* < 0.05) but equivalent to those with CpG alone (*P* > 0.05). This may have been due to the single immunization typically used with HA compared to prime/boost regimen with HBsAg and OVA. When IgG isotypes were measured as an indication of Th bias of responses (IgG2a or 2c indicative of Th1 and IgG1 indicative of Th2), IgG responses were predominantly Th1 biased with CpG, whether alone or combined with ISCOMATRIX. With ISCOMATRIX alone, the IgG2/IgG1 ratio was different for each antigen, indicating that antigen itself can play a role in determining Th bias of immune responses (Figures 1, 2, and 3).

### 3.2. Effect of Different Adjuvants on Antigen-Specific CTL Activity

**Figure 4:**
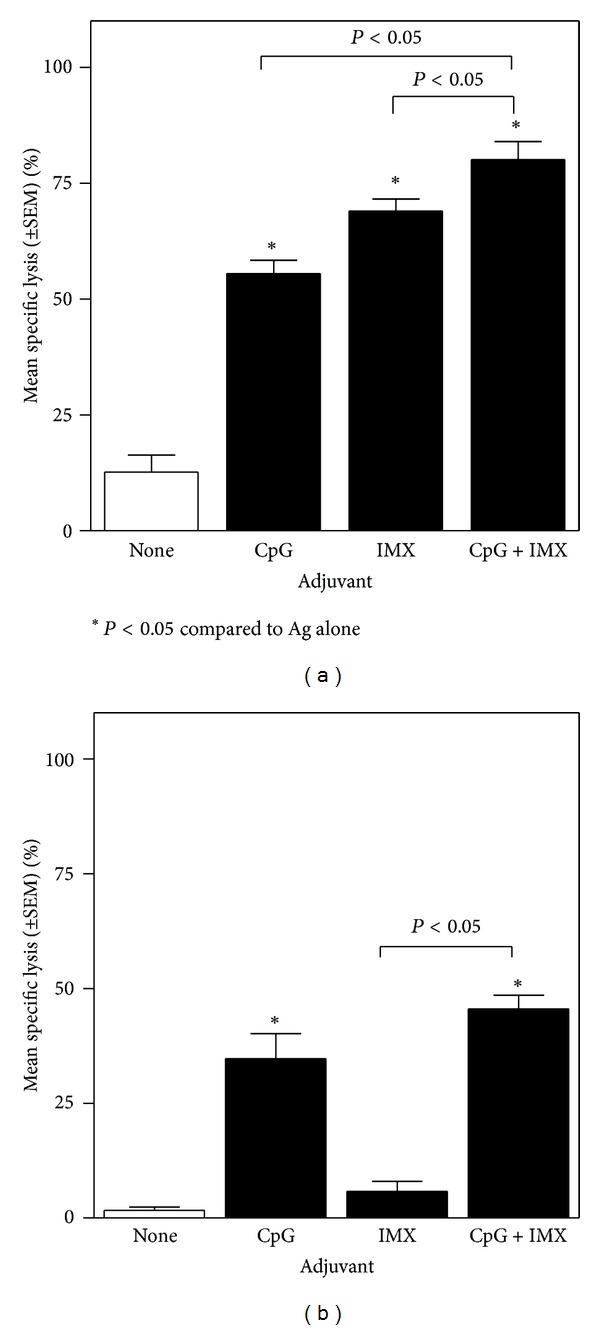
(a) BALB/c mice (*n* = 10/group) were immunized on days 0 and 28 by IM injection of 1 *μ*g HBsAg either alone (none) or combined with CpG (10 *μ*g), ISCOMATRIX (IMX) (1 *μ*g), or CpG (10 *μ*g) + ISCOMATRIX (1 *μ*g). Spleens were removed 4 wks after second immunization and CTL activity was determined. Bars represent the HBsAg-specific lysis as a percentage of the total possible lysis (% specific lysis) at an effector : target ratio of 50 : 1. (b) C57Bl/6 mice (*n* = 10/group) were immunized on days 0, 14, and 21 by IM injection of 10 *μ*g OVA either alone (none), or combined with CpG (10 *μ*g), ISCOMATRIX (1 *μ*g), or CpG (10 *μ*g) + ISCOMATRIX (1 *μ*g). Spleens were removed 1 wk after final immunization and CTL activity was determined. Bars represent the OVA-specific lysis as a percentage of the total possible lysis (% specific lysis) at an effector : target ratio of 50 : 1. The percent lysis was calculated as [(experimental release − spontaneous release)/(total release − spontaneous release)] × 100. Spontaneous release was determined by incubating target cells without effector cells, and total release was determined by adding 100 *μ*L of 2 N HCl to the target cells. The percent specific lysis was calculated as follows: % lysis with Ag-expressing cells − % lysis with control cells. Representative data from one of five independent experiments is shown in each graph.

Only very low CTL activity was measured with HBsAg or OVA alone (Figure 4). With single adjuvants, CpG induced strong CTL activity with both antigens (*P* < 0.05 compared to Ag alone), whereas ISCOMATRIX induced strong CTL activity with HBsAg but not with OVA (*P* < 0.05 and *P* > 0.05, resp.). The CpG/ISCOMATRIX combination further augmented CTL responses compared to either adjuvant alone with HBsAg (e.g., *P* < 0.05 at E : T of 50 : 1) but not with OVA (*P* > 0.05). 

Overall, CTL activity was lower with OVA antigen compared to HBsAg. HBsAg, a virus-like particle, can use alternate MHC1 presentation pathways that could result in more CD8^+^ IFN-*γ*-producing cells than would be produced with a soluble protein such as OVA. While our results suggest this may be the case, it was not within the scope of the current study to further investigate this. Responses were shown to be antigen specific, since only very low CTL activity was measured in samples that were stimulated with control cells which did not express antigen (results not shown).

### 3.3. Effect of Different Adjuvants on Antigen-Specific IFN-*γ* Secretion

**Figure 5:**
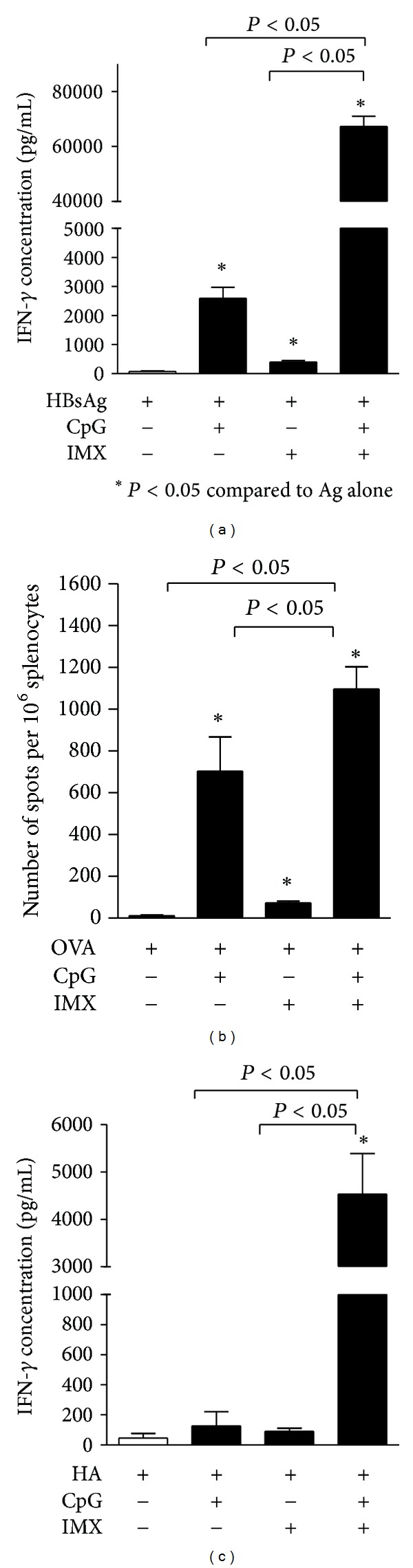
(a) BALB/c mice (*n* = 10/group) were immunized on days 0 and 28 by IM injection of 1 *μ*g HBsAg either alone (none) or combined with CpG (10 *μ*g), ISCOMATRIX (IMX) (1 *μ*g), or CpG (10 *μ*g) + ISCOMATRIX (1 *μ*g). Spleens were removed 4 wks after second immunization and IFN-*γ* secretion was measured by ELISA in antigen-restimulated splenocytes culture supernatants. (b) C57Bl/6 mice (*n* = 10/group) were immunized on days 0, 14, and 21 by IM injection of 10 *μ*g OVA either alone (none) or combined with CpG (10 *μ*g), ISCOMATRIX (1 *μ*g), or CpG (10 *μ*g) + ISCOMATRIX (1 *μ*g). Spleens were removed 1 wk after final immunization and IFN-*γ* secretion was measured by ELISPOT in antigen-restimulated splenocytes culture supernatants. (c) BALB/c mice (*n* = 10/group) received a single IM immunization of 1 g influenza. A haemagglutinin antigen (HA) either alone (none) or combined with CpG (10 *μ*g), ISCOMATRIX (1 *μ*g) or CpG (10 *μ*g) + ISCOMATRIX (1 *μ*g). Spleens were removed 4 wks after immunization and IFN-*γ* secretion was measured by ELISA in antigen-restimulated splenocytes culture supernatants. Representative data from one of five independent experiments is shown for HBsAg and OVA and one of two independent experiments for HA in each graph.

Only very low levels of IFN-*γ* were detected in supernatants from mice immunized using antigen alone (Figure 5). CpG alone enhanced IFN-*γ* levels with both HBsAg and OVA (*P* < 0.0001) but not with HA, whereas ISCOMATRIX alone induced only low levels of IFN-*γ* with all three antigens. The CpG/ISCOMATRIX combination enhanced IFN-*γ* secretion with all three antigens (*P* < 0.05), although there was a particularly strong synergy with HBsAg and HA which had IFN-*γ* concentrations 20- to 30-fold higher than with CpG or ISCOMATRIX alone (*P* < 0.05). For example, using HBsAg as an antigen, mean IFN-*γ* levels were approximately 2600, 400, and 70000 pg/mL with CpG, ISCOMATRIX, or the CpG/ISCOMATRIX combination, respectively, indicative of a synergistic rather than additive effect (Figure 5(a)). Likewise, using HA as an antigen, mean IFN-*γ* levels were approximately 125, 100, and 5000 pg/mL with CpG, ISCOMATRIX, or the CpG/ISCOMATRIX combination, respectively, (Figure 5(c)). Two different methods of measuring IFN-*γ* were employed (i.e., ELISA or ELISPOT) due to reagent availability at the time of each study; however, data obtained with different assays were not directly compared. Nevertheless, with both methods a strong synergy in IFN-*γ* secretion was observed with the CpG/ISCOMATRIX combination compared to either adjuvant alone. Responses were shown to be antigen specific, since only low levels of IFN-*γ* were obtained in samples that were not stimulated with antigen (results not shown). For all experiments, IFN-*γ* levels in response to ConA stimulation were equivalently strong (data not shown).

Only very low levels of IL-4 were detected in supernatants from mice immunized using any of the antigens and there was no increase seen in the presence of adjuvants (data not shown).

### 3.4. Evaluation of Different Adjuvants in B-16 Melanoma Model

**Figure 6:**
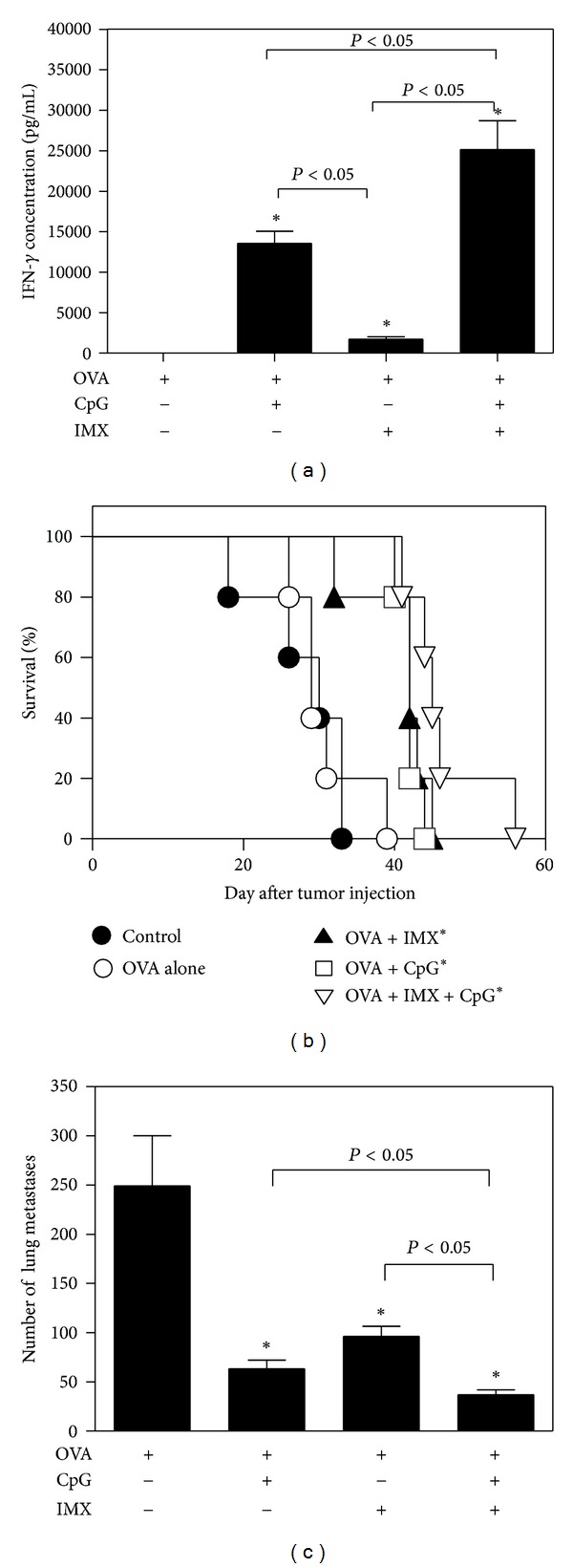
C57Bl/6 mice (*n* = 10/group) received 5 × 10^5^ live B16 melanoma cells expressing ovalbumin by IV administration in order to establish tumors. Control animals received PBS. On days 7 and 14 after tumor cell administration, mice were immunized by IM injection with 50 *μ*g OVA alone or in combination with CpG (10 *μ*g), ISCOMATRIX (IMX) (1 *μ*g), or CpG (10 *μ*g) + ISCOMATRIX (1 *μ*g). On day 21 after tumor challenge, splenocytes from five animals per group were harvested. Remaining animals were monitored for survival. Panel (a): each bar represents IFN-*γ* secretion measured by ELISA in antigen-restimulated splenocyte culture supernatants. Panel (b): survival over time is shown for each treatment group. Panel (c): each bar represents the number of lung metastases counted per treatment group at 21 days after tumor induction. Representative data from one of two independent experiments is shown. *P < 0.05 compared to Ag alone.

On day 21 after tumor induction, five animals from each treatment group were euthanized, and IFN-*γ* secretion was measured following antigen restimulation of splenocytes. Only very low levels of IFN-*γ* were detected in supernatants from mice immunized using OVA alone (Figure 6(a)). Both CpG and ISCOMATRIX alone enhanced IFN-*γ* secretion compared to antigen alone (*P* < 0.05), with significantly higher levels of IFN-*γ* obtained in supernatants from mice immunized using CpG alone as an adjuvant compared to ISCOMATRIX alone (*P* < 0.05). Significantly higher levels of IFN-*γ* were obtained in supernatants from mice immunized using the CpG/ISCOMATRIX combination compared to when either was used alone (*P* < 0.05).

### 3.5. Effect of CpG/ISCOMATRIX Adjuvant Combination on Survival and Metastasis

The effect of immunization on survival and lung metastasis was measured following administration of the murine tumor model, B16-OVA melanoma. All nonimmunized control animals died or were euthanized for humane reasons by day 33 after inoculation of tumor cells (Figure 6(b)), and upon necropsy all control animals were found to have developed extensive lung metastases which were too numerous to count (>300) (data not shown). Immunization of animals with OVA alone did not significantly enhance survival or reduce metastasis compared to control animals (*P* > 0.05), such that by day 40 all animals had died or were euthanized for humane purposes. Immunization using CpG or ISCOMATRIX alone significantly enhanced survival and reduced metastasis over OVA alone or placebo controls (*P* < 0.05) (Figure 6). Median survival time was 30 days for unimmunized control animals and 29, 42, 42, and 45 days for animals immunized with OVA alone, OVA + CpG, OVA + ISCOMATRIX, or OVA + CpG/ISCOMATRIX combination, respectively. Therefore, for example, on day 40 when 100% of animals immunized with antigen alone had succumbed to disease, mortality was only 0 to 20% for animals immunized using ISCOMATRIX, CpG, or the CpG/ISCOMATRIX, with no significant differences in longevity between these groups. Although all animals in these groups eventually succumbed to disease, those immunized with OVA and CpG/ISCOMATRIX combination had significantly reduced lung metastasis compared to either adjuvant alone (*P* < 0.05) (Figure 6(c)). 

### 3.6. Comparison of Effects of CpG/ISCOMATIX and CpG/Alum Adjuvant Combinations on HBsAg-Specific IFN-*γ* Secretion

**Figure 7:**
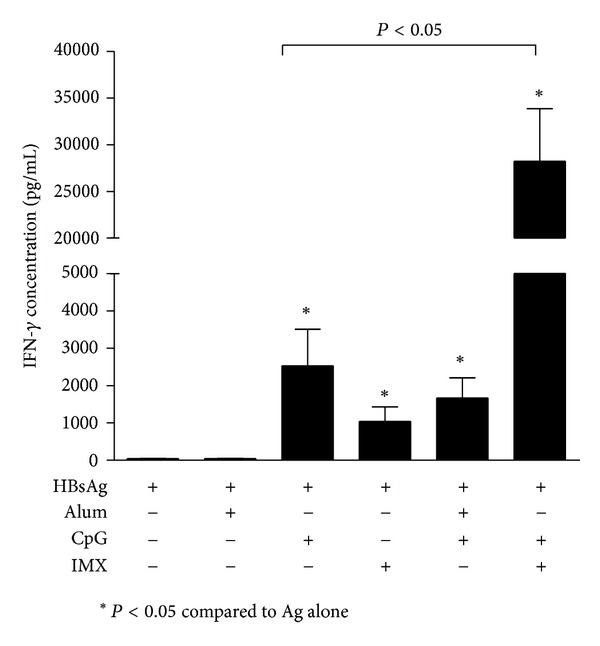
BALB/c mice (*n* = 10/group) were immunized on days 0 and 28 by IM injection of 1 *μ*g HBsAg either alone (none) or combined with CpG (10 *μ*g), ISCOMATRIX (IMX) (1 *μ*g), aluminum hydroxide (alum) (25 *μ*g Al^3+^), CpG (10 *μ*g) + aluminum hydroxide (alum) (25 *μ*g Al^3+^), or CpG (10 *μ*g) + ISCOMATRIX (1 *μ*g). Spleens were removed 4 wks after the second immunization and IFN-*γ* secretion was measured by ELISA in antigen-restimulated splenocytes culture supernatants.

The CpG/ISCOMATRIX adjuvant combination enhanced HBsAg-specific IFN-*γ* secretion compared to either adjuvant alone (*P* < 0.05), whereas levels obtained with CpG/alum were equivalent to those obtained with CpG alone (*P* > 0.05). Mean IFN-*γ* levels obtained with the CpG/ISCOMATRIX combination were approximately 15-fold higher than those obtained with the CpG/alum adjuvant combination (Figure 7).

## 4. Discussion

For the development of effective antitumor or antiviral disease vaccines, strong immune adjuvants are required to induce robust immune responses. Herein, we have compared CpG with ISCOMATRIX using different antigens and adjuvant combinations. We have shown that both CpG and ISCOMATRIX are strong adjuvants when used alone, which was not surprising, as both CpG and ISCOMATRIX have successfully been used as adjuvants in multiple species, including humans [16, 25]. The strong adjuvanticity associated with CpG and ISCOMATRIX could be further enhanced when used in combination. In particular, a strong synergy was seen in IFN-*γ* secretion with all three antigens despite the fact that different immunization protocols were used with each antigen. Overall, the strongest synergy was seen with HBsAg which had the longest time interval between doses (4 weeks). Since immunization schedule can impact both strength and nature of induced responses, it is possible that responses with OVA and HA as antigens could be further improved with either an increased time interval between doses (OVA) or an increased number of doses (HA). 

The strong adjuvanticity seen in this study associated with the combination of CpG and ISCOMATRIX is in line with previous reports in mice using immunostimulatory complexes (ISCOMs). For example, immunization with inactivated *Francisella tularensis* live vaccine strain (LVS) adjuvanted with CpG combined with preformed ISCOMs provided better protection than an alum-adjuvanted vaccine when challenged with a virulent strain of *F. tularensis* [26, 27]. Also, immunization with cytomegalovirus (CMV) glycoprotein B vaccine formulated with CpG and ISCOMs elicited strong CMV-specific immunity against multiple CMV strains [28]. To evaluate whether the strong synergy between CpG and ISCOMATRIX seen in immunological readouts would translate to better functionality, we also evaluated the CpG/ISCOMATRIX combination in a therapeutic metastatic lung carcinoma model. In this model, the CpG/ISCOMATRIX combination also induced significantly higher levels of OVA-specific IFN-*γ* secretion compared to either adjuvant alone, and this translated into a reduction in lung metastases in these animals. However, despite the strong antitumor immune responses, all animals eventually succumbed to the cancer. It is possible that the kinetics and/or strength of the immune response were not optimal or fast enough to curtail an already established tumor, that an immune response against a single tumor antigen is not sufficient to control tumor growth, or that although we could measure OVA-specific T cell responses ex vivo, these cells may have been rendered inactive at the site of the tumor by immune suppressive mechanisms induced by the tumor. Nevertheless, although there was no long-term survival benefit, the CpG/ISCOMATRIX combination did help induce strong enough immune responses to significantly reduce lung metastasis in this aggressive tumor model. 

Similar results have been demonstrated in a murine pancreatic carcinoma model, whereby the inclusion of CpG in an ISCOM-based vaccine was shown to reduce numbers of regulatory T cells, enhance CTL responses, and induce regression of pancreatic tumors [29]. It is possible that we would have seen a stronger synergy in our tumor challenge studies with additional boosts or a different immunization schedule; however, the very aggressive nature of this tumor model made this difficult to evaluate. It should also be noted that the adjuvant dose when used in combination was not optimized but rather used at the optimal dose for each adjuvant alone. It is possible that changing the dose of either adjuvant may produce better responses as shown in the pancreatic carcinoma studies. There are almost endless combinations of doses that are possible and doing such studies in mouse models is of limited value as the optimal human dose for each adjuvant alone is quite different from that in mice.

The increased immunogenicity seen with the CpG/ISCOMATRIX combination is likely due to the immunomodulatory properties of the adjuvant mixture as well as enhanced delivery and/or protection from degradation of the CpG by ISCOMATRIX. Indeed, for optimal synergy between CpG and ISCOMATRIX both the delivery components (phospholipids and cholesterol) and immunomodulatory components (ISCOPREP saponin) of ISCOMATRIX are required, since when CpG were combined with ISCOPREP saponin alone, although stronger secretion of IFN-*γ* was observed than with either adjuvant alone, levels of IFN-*γ* were lower than those observed with the CpG/ISCOMATRIX combination (data not shown). Although the exact mechanism of action of ISCOMATRIX has not yet been fully elucidated, it has recently been shown to combine both immune activation as well as enhanced antigen delivery to DCs leading to an effective cross-priming ofCD8^+^T cells [21, 30]. The uptake of antigen when formulated with ISCOMATRIX by DCs occurs via endocytosis with delivery of antigen to lysosomes and subsequent rapid translocation to the cytosol resulting in enhanced Ag cross-presentation [30]. It is possible that this efficiency of delivery to the endosome of molecules also applies to CpG and may play a role in the strong synergy seen with the combination, since the CpG receptor TLR9 is located in the endosomal compartment [31], and CpG-mediated activation of this receptor can enhance antigen cross-presentation [32]. The direct delivery of substances to the endosome is likely critical to the strong synergistic effect seen with the CpG/ISCOMATRIX combination, since alum, another delivery system, failed to achieve the same result. 

Therefore, the significant enhancement in antigen-specific IFN*γ* secretion observed with the CpG/ISCOMARIX combination may result from enhanced antigen cross-presentation mediated by both adjuvants. It is also known that both adjuvants activate innate immune cells and so a combined effect may lead to a much more optimal local environment for the adaptive immune response to occur. As ISCOMATRIX does not contain TLR ligands it is highly unlikely that it signals through TLRs as supported by a number of in vitro studies (data not shown). It has been shown, however, that the MyD88 signaling pathway is important for the induction of strong IFN^+^ CD8^+^ T cell responses with ISCOMATRIX vaccines so there does appear to be some overlap with CpG signaling pathways [22]. 

Ultimately, as with all animal studies, limitations exist for extrapolation in humans and it remains to be determined whether CpG and ISCOMATRIX will prove to be a useful adjuvant combination in the clinic. It is possible that activity in humans may be less than in mice, due to differences in TLR9 expression in human and mouse dendritic cell populations, although a number of studies have now shown that CpG are still active in higher species, including humans. 

## 5. Conclusions

The studies presented herein highlight that the rational combination of different adjuvants could lead to the development of novel vaccines or the enhancement of existing vaccines. Vaccination strategies that include both CpG and ISCOMATRIX are one means by which the strong Th1 immune responses required to prevent and treat chronic infection and tumors may be achieved.
